# Grade 3 Radiation Recall Sigmoiditis after Treatment for Locally Advanced Cervical Cancer: A Case Report

**DOI:** 10.7759/cureus.353

**Published:** 2015-10-15

**Authors:** Audrey Tetreault-Laflamme, Francois Bachand

**Affiliations:** 1 Radiation Oncology, BC Cancer Agency, Sindi Ahluwalia Hawkins Centre for the Southern Interior

**Keywords:** radiation recall, sigmoiditis, cervical cancer

## Abstract

We report a case of Grade 3 radiation recall sigmoiditis after administration of a combination of carboplatin, paclitaxel, and bevacizumab, following irradiation for a locally advanced cervical cancer. A 50-year-old woman was diagnosed with an FIGO Stage IIIb squamous cell carcinoma of the cervix with bilateral pelvic and para-aortic lymph nodes. She underwent concurrent chemoradiation followed by high dose rate (HDR) intracavitary brachytherapy. She had a complete loco-regional response. A supraclavicular recurrence was diagnosed three months after completing treatment and two cycles of carboplatin, docetaxel, and bevacizumab were given in April 2014. Shortly after the second cycle, she was admitted to the hospital for significant abdominal pain, diarrhea followed by symptoms of bowel subocclusion. The CT scan and endoscopic images revealed thickening of the sigmoid wall with important edema and telangiectasia. The biopsy was consistent with acute radiation-induced colitis. Because of persistent digestive symptoms, a diverting ileostomy was done few months later. The location, timing, pathology, and its association with a high-dose region are analyzed in this case report.

## Introduction

Chemotherapy-induced radiation recall is an acute inflammatory reaction within a previously irradiated area triggered by pharmacologic agents. Its physiopathology is still unknown. Although infrequent and usually moderate, radiation recall phenomenon can lead to severe complications. This is a case of a Grade 3 radiation recall sigmoiditis after the administration of a combination of carboplatin, paclitaxel, and bevacizumab four months post-pelvic irradiation for locally advanced cervical cancer, requiring diverting ileostomy. The patient consent for publication was obtained prior to this report.

## Case presentation

We report the case of a 50-year-old woman, otherwise known for active smoking and hypertension, presenting with FIGO Stage IIIb squamous cell carcinoma of the cervix with bilateral pelvic and para-aortic lymph nodes in 2013. Pelvic examination, MRI, and PET-CT revealed a 6.3 x 7.6 x 8.1 cm cervical mass with bilateral parametrial involvement, fixed to the pelvic wall bilaterally. There was evidence of extensive FDG-avid lymphadenopathy extending from the external iliac veins bilaterally up to the left renal vein without distant metastasis.

Concurrent chemoradiotherapy was given with weekly cisplatin at a dose of 40 mg/m^2^ between October 1 and November 29, 2013. She received 45 Gy in 25 fractions of IMRT external beam radiotherapy to the pelvic and whole para-aortic region followed by a boost of 9 Gy in five fractions delivered to the FDG-avid lymph nodes. MRI-based (HDR) combined intracavitary and interstitial brachytherapy delivered 30 Gy over five fractions to the high-risk clinical target volume (CTV). The treatment was completed within eight weeks. She developed expected mild side effects of pelvic irradiation, mostly diarrhea, that had resolved by January, 2014. 

The first PET-CT scan, three months after treatment, revealed a single 1 cm FDG-avid left supraclavicular lymph node while showing a complete response in the pelvis and abdomen. A nodal biopsy confirmed metastatic squamous cell carcinoma of cervical origin. Chemotherapy was started in April, 2014 (four months after initial treatment) using a combination carboplatin (AUC 5), paclitaxel (175 mg/m^2^), and bevacizumab (15 mg/kg) every three weeks.

**Figure 1:**
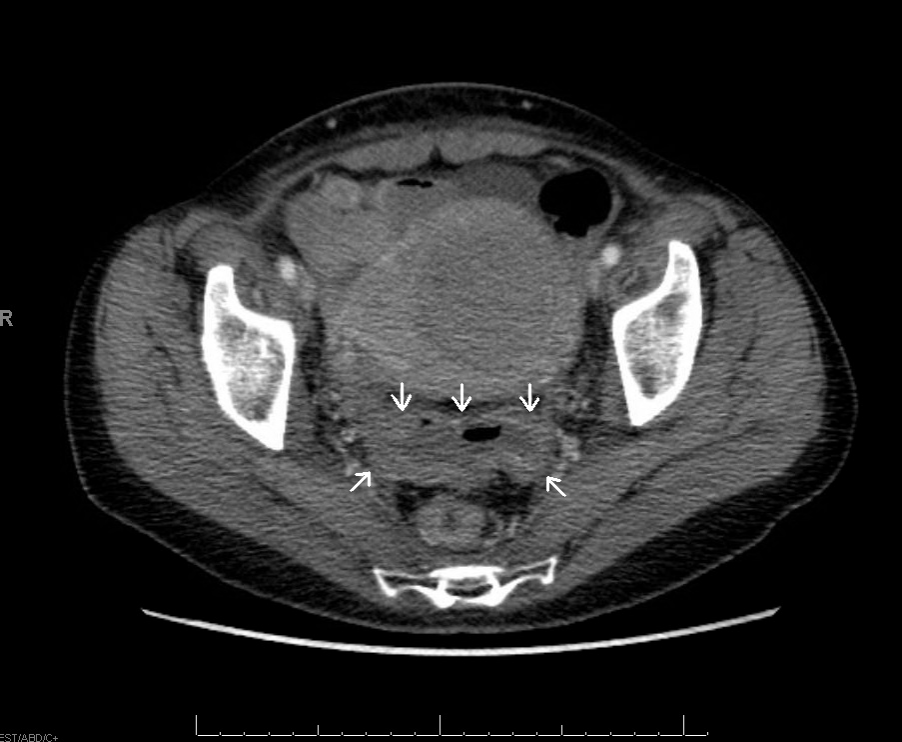
CT-scan transverse image showing thickening of the sigmoid wall

Shortly after the second cycle of chemotherapy, she developed significant abdominal pain, nausea, vomiting, and diarrhea, requiring admission. A CT scan in May, 2014 showed a 1 cm-mural thickening of the mid sigmoid with a small amount of free fluid. There was no obstruction, perforation, or abscess seen, nor evidence of recurrent disease in the abdomen (Figure 1). The sigmoidoscopy revealed edema and telangiectasia of the rectosigmoid, positive for acute, focally erosive colitis on biopsy. The findings were consistent with radiation-induced damage, and there was no evidence of granulomatous inflammation, dysplasia, or malignancy. She was treated with hydration, antibiotics, and supportive care and discharged two months later.

Recurrent episodes of bowel subocclusion, abdominal pain, and cramps led to an elective diverting loop ileostomy in September, 2014. The patient recovered rapidly after surgery, gaining weight, and recovering normal bowel control. With one year of follow-up, there are no signs of recurrent disease in the pelvis, abdomen, and supraclavicular fossa.

## Discussion

Radiation recall is a rare, acute inflammatory reaction, which occurs in a previously irradiated area, induced by the administration of pharmacologic agents. It was first described following the use of chemotherapy agents, but also reported with other classes of drugs, such as antibiotics, tamoxifen, and targeted therapies [1-12]. The most frequent reaction reported is radiation recall dermatitis with various grades of reaction from mild erythema to severe ulceration [4, 13-14]. Other organs can also be affected, including the lungs, mucosa, and digestive tract [15]. There are few reported cases of radiation recall colitis [1-2, 16]. Kundak, et al. published the case of a 63-year-old woman, previously irradiated for cervical cancer, who developed radiation colitis following the administration of paclitaxel and carboplatin for multiple lung metastases. She presented with diarrhea and rectal bleeding after the administration of each course of chemotherapy and was treated with supportive care every time [3]. 

**Figure 2:**
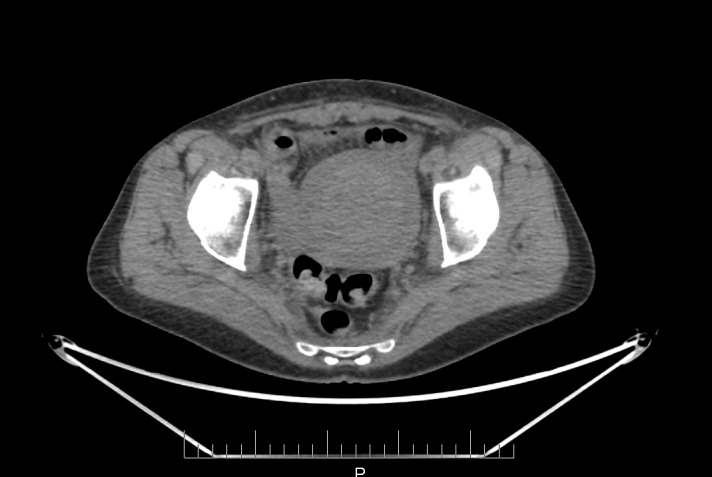
CT scan transverse image from the PET-CT showing normal thickness of the sigmoid, 3 months post-radiation

Our patient was not known for colitis prior to her treatment and did not have symptoms suggesting an underlying disease. All acute side-effects had completely resolved after radiotherapy. The CT scan from the PET-CT, three months post-radiation, showed a normal thickness of the sigmoid wall without evidence of colitis (Figure 2). Four months after completing radiotherapy and immediately following the second cycle of chemotherapy, she developed acute symptoms, clinical and radiological signs of acute colitis. The main differential diagnosis would be late radiation-induced colitis. However, considering that the symptoms and signs were acute, that there were no visible changes on CT at three months, and that it would be deemed early for late toxicity from treatment, the most likely diagnosis remained chemotherapy-induced radiation recall reaction.

**Figure 3:**
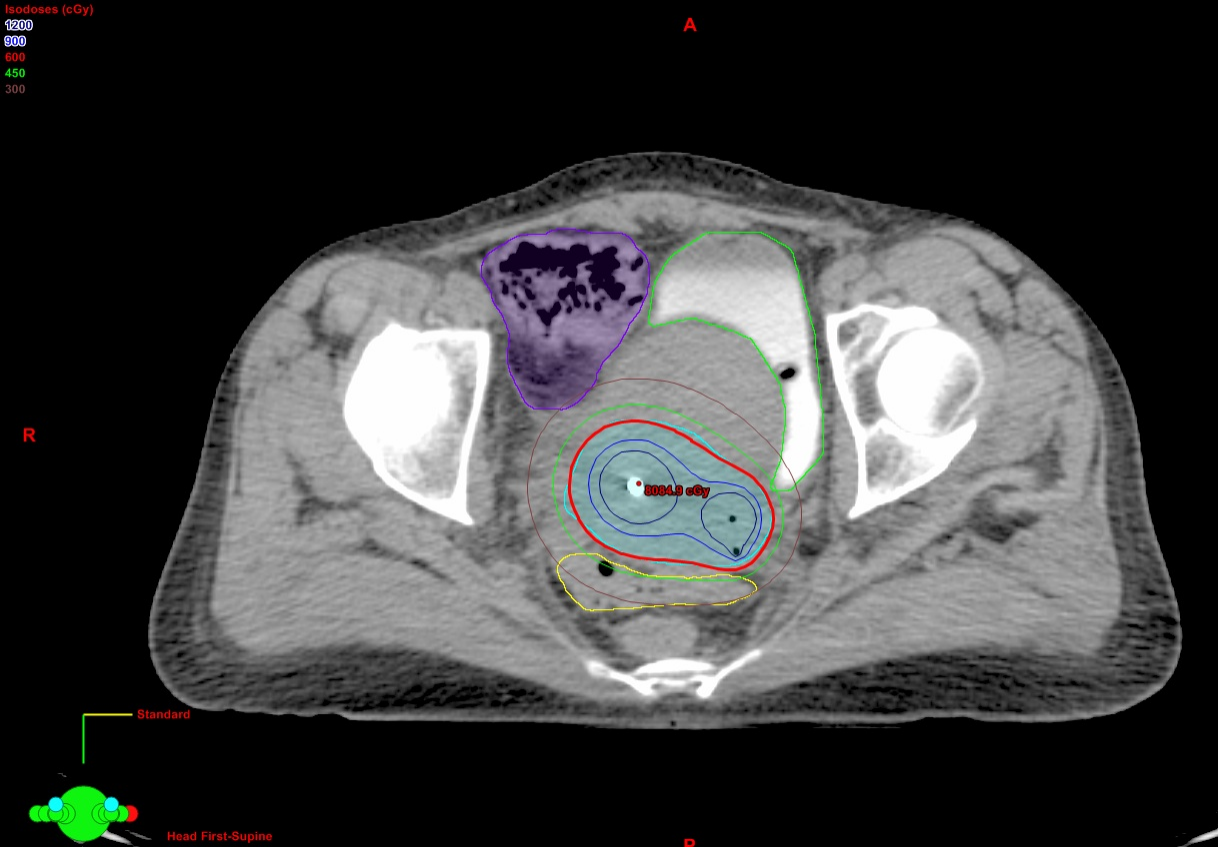
Transverse view of the high-dose region from brachytherapy. Sigmoid (in yellow) was adjacent to the HR-CTV (in cyan) .

The CT scan specifically shows thickening of the sigmoid that was localised just posterior to the cervix and the uterus, corresponding to the high-dose region from brachytherapy (Figures 1, 3). The dosimetry from brachytherapy and pelvic irradiation were reviewed using the “worst case” hypothesis, assuming that the high-dose region from brachytherapy was the same for each fraction and that it also received the whole pelvic dose. Equivalent doses (EQD2) were used to calculate the minimum dose to the most irradiated 2 cm^3^ of sigmoid (D2cc), as per GEC-ESTRO recommendations [17]. Using those parameters, the total sigmoid D2cc was 73 Gy, which is slightly higher than the GEC-ESTRO recommendation (sigmoid D2cc ≤ 70 Gy) but acceptable considering the close proximity of the sigmoid to the high-risk CTV. Radiation recall phenomenon has been associated with a wide range of radiation doses from 10 to 81 Gy and no threshold has been identified [15].

In this specific case, the chemotherapy combination included bevacizumab, a monoclonal antibody that binds to the vascular endothelial growth factor (VEGF) receptor, thus inhibiting angiogenesis. Its ischemic effect could have contributed to the reaction or deteriorated an underlying colitis. A case of bevacizumab-induced radiation recall gastritis was described by Saif, et al. A 67-year-old woman was initially treated with concurrent chemoradiation followed by maintenance gemcitabine and bevacizumab for a locally advanced pancreatic adenocarcinoma. During the maintenance phase, she developed gastrointestinal bleeding due to gastric lesions consistent with radiation-induced changes. Bevacizumab was discontinued and the patient pursued with gemcitabine monotherapy with no recurrence of bleeding [12]. 

## Conclusions

Radiation recall is a rare, unpredictable, and still misunderstood phenomenon. The diagnosis of radiation recall reaction can be challenging and should be recorded carefully. We report a case of Grade 3 radiation recall sigmoiditis in the high-dose region from brachytherapy requiring diverting ileostomy after receiving a combination of carboplatin, paclitaxel, and bevacizumab.
